# Controlling Mast Cell Activation and Homeostasis: Work Influenced by Bill Paul That Continues Today

**DOI:** 10.3389/fimmu.2018.00868

**Published:** 2018-04-26

**Authors:** Heather L. Caslin, Kasalina N. Kiwanuka, Tamara T. Haque, Marcela T. Taruselli, H. Patrick MacKnight, Anuya Paranjape, John J. Ryan

**Affiliations:** ^1^Department of Biology, Virginia Commonwealth University, Richmond, VA, United States; ^2^Department of Biochemistry and Molecular Biology, Virginia Commonwealth University, Richmond, VA, United States

**Keywords:** mast cell, IgE, IgG, IL-4, IL-10, TGF-β, Stat5, allergy

## Abstract

Mast cells are tissue resident, innate immune cells with heterogenous phenotypes tuned by cytokines and other microenvironmental stimuli. Playing a protective role in parasitic, bacterial, and viral infections, mast cells are also known for their role in the pathogenesis of allergy, asthma, and autoimmune diseases. Here, we review factors controlling mast cell activation, with a focus on receptor signaling and potential therapies for allergic disease. Specifically, we will discuss our work with FcεRI and FγR signaling, IL-4, IL-10, and TGF-β1 treatment, and Stat5. We conclude with potential therapeutics for allergic disease. Much of these efforts have been influenced by the work of Bill Paul. With many mechanistic targets for mast cell activation and different classes of therapeutics being studied, there is reason to be hopeful for continued clinical progress in this area.

## Introduction

Mast cells were first described by Paul Ehrlich in 1878. The future Nobel Laureate identified them based on their unique staining characteristics with aniline dyes and their position in all tissues of the body, particularly at interfaces with the external environment (1). As with T cells and macrophages, mast cells are a heterogeneous population, consisting of at least two major subsets (2). Although the origin of these cells remained elusive, seminal work in the 1970s and 1980s established that mast cells originate from hematopoetic stem cells in the bone marrow, spleen, fetal liver, and peripheral blood (3–5). Subsequent work showed that mast cells can be differentiated and expanded *in vitro* with relative ease, which greatly increased interest and progress in the field. What followed was detailed work describing how mast cells bind and respond to IgE, providing evidence for the role of mast cells in allergic disease (6, 7).

However, our understanding of mast cell biology changed drastically in the late 1980s with work by Bill Paul and colleagues. Bill Paul’s career centered on understanding T cell function and cytokine biology, contributing to the discovery, and understanding of T cell MHC-restriction, the B cell receptor mIg, IL-4, and Th2 polarization, as he eloquently described in a review of his life’s work (8). Following the discovery of IL-4, Bill Paul’s group showed that transformed and non-transformed mast cells express IL-4 in response to PMA and ionomycin (9) and that mast cells secrete a Th2-like panel of cytokines, including IL-4, in response to IgE receptor cross-linking (10). These were tectonic shifts in our fundamental understanding of mast cells, providing evidence that in addition to granule release, mast cells produce cytokine mediators that influence adaptive immunity and have a broader role in allergic disease. It is in keeping with Bill Paul’s visionary abilities that he could abruptly cast a broad light on field tangential to his primary interests. He would go on to publish two dozen mast cell-related articles, including one that initiated our group’s focus on Stat5 in mast cell biology (11). Furthermore, Bill trained many researchers who have gone on to have productive careers in the field of mast cell biology and allergic disease, including the senior author of this article, Takashi Saito, Fred Finkleman, Melissa Brown, Achsah Keegan, and Joshua Milner, many of whom have work cited here. In this review, we will cover several areas of mast cell activation and homeostasis, all of which are of great interest to our lab and have been impacted by Bill Paul’s intellect and productivity.

## Mast Cell Growth, Survival, and Apoptosis

**Figure 1:**
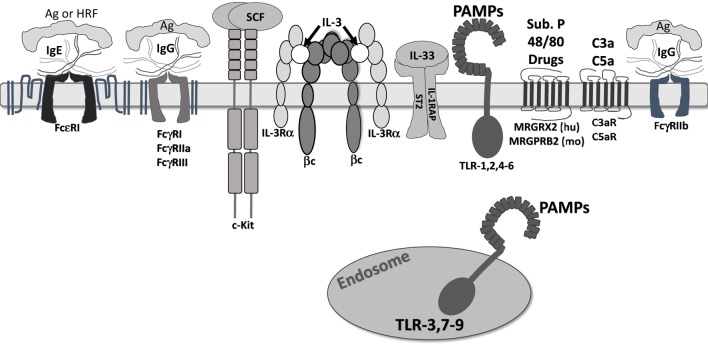
Receptors that regulate mast cell function. The receptors shown are confirmed to regulate mast cell function. They are depicted at approximate scale. All except FcγRIIb are known to induce mast cell degranulation and/or cytokine secretion. FcγRIIb activates SHIP-1 and SHP-1, suppressing inositol and tyrosine kinase activity. c-Kit is a weak mast cell activator, but augments signals by other receptors. IL-3 receptor is modeled after work by Broughton et al. (20). Note that ligands for Mas-related G protein-coupled receptor-X2 (MRGPRX2)/B2 are not fully known, but include drug classes discussed in the accompanying text. In addition, we do not show “cytokinergic” IgE molecules. These form aggregates in the absence of antigen and elicit FcεRI signaling.

Mast cells are long-living tissue-resident immune cells that migrate to and differentiate within the tissue. Development, migration, and survival are shaped by two growth factors, in particular, SCF and IL-3, which are included in Figure 1. In healthy tissue, mast cells are maintained in constant numbers, while the mast cell population increases dramatically in chronically allergic tissue (12). This section will summarize findings on mast cell survival and death. Prior to discovery of the c-Kit receptor and its ligand SCF, mice with double mutations at the *ckit*-encoding *W* loci (W/W^v^ mice) or *scf*-encoding *Sl* loci (Sl/Sl^d^ mice) were known to exhibit hypoplastic, macrocytic anemia, sterility, and a lack of cutaneous melanocytes (13–15). Importantly, these mice were found to have a defect of mast cells in W/W^v^ mice due to lineage abnormality and a defect of mast cells in Sl/Sl^d^ mice due to an abnormality in the microenvironment (4, 16). A decade later, two groups reported that the *W* gene product encodes the c-Kit tyrosine kinase receptor (17, 18), while in 1990, eight groups described and identified the ligand for c-Kit: SCF/MGF/steel factor, encoded by the *SI* locus [prefaced in Ref. (19)]. These papers clarified the complementary receptor–ligand relationship yielding the similar phenotypes of W/W^v^ and Sl/Sl^d^ mice and suggested a role for c-Kit and SCF in mast cell development.

c-Kit is a tyrosine kinase growth factor receptor, with a large extracellular domain of five Ig-like domains, a single transmembrane span, and a long cytosolic tail, containing a tyrosine kinase domain and tyrosine phosphorylation sites (21). Dimerization initiates phosphorylation of at least eight tyrosine residues, serving as a docking site for Src homology 2 domains present on signaling proteins such as Grb2, Gab2, Lyn, Fyn, PI3K, phospholipase Cɣ, and the negative regulator, SHP-1 (22).

SCF is best known for eliciting mast cell survival and inhibiting apoptosis (23). SCF-mediated activation of the PI3K–AKT cascade is important for mast cell survival, inactivating the pro-apoptotic proteins Bad and Bim, and increasing expression of the pro-survival proteins, such as Bcl-2 and Bcl-XL (24, 25). Interestingly, somatic and germline *ckit* gain-of-function mutations are present in mastocytosis patients (26, 27), suggesting regulatory control by the c-Kit pathway.

In addition to its role in survival, SCF has other important effects. These include inducing mast cell migration, adhesion, and IL-6 secretion (28–30). SCF also augments mast cell activation by FcεRI, ST2, and TLR4, receptors which will be further reviewed below (31–33). c-Kit signaling enhances mast cell degranulation and cytokine production by inducing calcium influx and transcriptional activity (34). These abilities make SCF arguably the most critical factor controlling mast cell biology.

IL-3 is also an important factor for mouse mast cell precursor survival, with an unclear role in human mast cells. Mouse mast cells can differentiate in response to IL-3 (mucosal tissue type) or IL-3 + SCF (connective tissue type), while human mast cells typically require SCF + IL-6 for differentiation (35–38). Contradictory studies suggest that IL-3 plays no role in human mast cell progenitor differentiation (38), while a recent study demonstrated that IL-3 alone is sufficient to drive differentiation and survival of human mast cell progenitors (39). Similar to SCF, withdrawal of IL-3 from cultured mast cells induces apoptosis and appears to play an important role in mast cell survival and development in at least mouse mast cells (23, 40). Lantz et al. showed that IL-3-deficient mice have normal numbers of mast cells in the naïve state, but fail to properly expand intestinal mast cells in response to parasite infection (41).

To balance cell growth, mast cells have pathways for both apoptosis and autophagy. The Fas and TRAIL death receptors are expressed on primary mouse and human mast cells and various mast cell lines (42, 43). Mast cells are susceptible to Fas- or TRAIL-mediated apoptosis *via* caspase activation (1, 43). However, they can overcome Fas death signals by upregulating Fas-associated death domain-like IL-1-converting enzyme-inhibitory protein, a caspase-8 inhibitor that lacks the cysteine domain (42). While transformed and healthy mast cells use this mechanism to bypass Fas-mediated cell death, making this an unreliable and weak apoptosis inducer (43), our group has shown that that BMMCs are more responsive to Fas/FasL-induced apoptosis in the presence of IL-4 and IL-10 (44). Furthermore, rather than acting solely as an apoptosis signal, Fas functions in mast cell development and maturation (45).

In addition to apoptosis, mast cells also undergo autophagy, which controls the clearance and reuse of intracellular organelles and proteins and is essential for eukaryotic cell survival. Light chain (LC)-3 is localized to autophagosomes through conversion of LC3-I to LC3-II, which requires *Atg5* and *-7*. LC3-II-expressing autophagosomes are delivered to lysosomes, where the auto-cargo is degraded (46). Conversion of LC3-I to IC3-II has been shown to be constitutive in BMMC, in which LC3-II associated with secretory granules (47). The same study showed that BMMC lacking *Atg7* or *12* have normal granule formation, but defective IgE-mediated degranulation, demonstrating the importance of autophagic machinery in granule movement and release. Furthermore, dysregulation of autophagy in mast cells has been shown in various disease states such as systemic sclerosis, chronic rhinosinusitis, and asthma (48–50). Overall, research on mast cell survival, apoptosis, and autophagy pathways suggests that these pathways are important for both maintenance and function in both health and disease.

## Activating Ligands and Receptors Regulating Mast Cell Function

Mast cells respond to myriad signals consistent with their role in defense against pathogens, while contributing to their effects in allergy, asthma, and autoimmunity. In addition, mast cells are a heterogeneous population, particularly susceptible to different tissue microenvironments during development and maturation. Residence in different tissues is linked to distinct mast cell protease and receptor expression (51–53). Moreover, mast cell phenotype is highly “tunable” based on short-term modulation by inflammatory stimuli, growth factors, cytokines, and metabolites (52, 54–56). We will first describe several activating receptors and their stimuli, which are depicted in Figure 1.

### IgE and FcεRI

FcεRI, the receptor for IgE, has been the most commonly studied mast cell receptor. While new studies suggest a role for many mediators in allergic disease, IgE remains the best-understood mechanism of mast cell activation in allergic disease (57). The interaction between mast cells and IgE was first shown in 1970 (6, 7). A previously unknown component found in the serum of allergic patients, IgE played a role in the classic Prausnitz–Küstner reaction. In addition, these studies showed that IgE bound to mast cells and basophils. Further characterization found the interaction between IgE and FcεRI, showing that monomeric IgE binds to a singular unit of FcεRI with very high affinity (58). Subsequent studies showed that FcεRI consists of three subunits: one IgE-binding α subunit, one β subunit, and a dimer of disulfide-linked γ subunits (59). FcεRI-mediated activation typically occurs when IgE, bound by its Fc portion (specifically the C_H_3 domain), interacts with antigen *via* the Fab portion, driving receptor aggregation. This initiates signaling cascades dependent on tyrosine phosphorylation, leading to a biphasic response. The first phase is the immediate degranulation. The second phase is the production of other mediators such as prostaglandins, leukotrienes, cytokines, chemokines, and growth factors (60).

Many of the pathways controlling FcεRI-induced mast cell activation have been described in detail, and we direct the reader to other recent reviews (61–63). However, some aspects of FcεRI activation warrant special attention here. In addition to signaling induced by receptor aggregation, monomeric IgE induces mast cell survival and activation (64–66). Prior to this, there was knowledge that IgE greatly increases FcεRI expression (67, 68), but its ability to induce signals in the absence of antigen was unexpected and controversial. Subsequent studies revealed that the clone of IgE molecule used greatly impacted its effects, with different IgE clones being designated “highly cytokinergic” or “poorly cytokinergic.” The latter type has since been shown to form large aggregates through Fv–Fv interactions in the absence of antigen, and hence signals much like IgE + antigen (69). In a similar way, histamine-releasing factor (also called translationally controlled tumor protein or fortillin) binds the Fab region on approximately 25% of IgE molecules tested, allowing for clonally restricted mast cell activation that may be more common among atopic patients (59). These unexpected and clinically important effects of IgE subtypes emphasize the importance of fundamentally understanding receptor–ligand interactions. They also support the approach of suppressing IgE–FcεRI interaction, which is proving effective with the drug omalizumab, discussed in the “Potential Therapies” section below.

### IgG and FcγR

Mast cells can be activated by IgG immune complexes binding pro-inflammatory FcγRI, FcγRIIA, or FcγRIII, which are variably expressed on mouse and human mast cells. These receptors induce a signaling cascade resembling IgE–FcεRI activation that elicits cytokine secretion, arachidonic acid metabolism, and degranulation (70). Our lab has published work suggesting that like FcεRI, FcγR induces Fyn, Lyn, Akt, Erk, p38, and JNK phosphorylation (71–73). Opposingly, the ITIM-containing receptor FcγRIIb activates SHIP-1 and SHP-1, suppressing IgG-induced signals by reducing PI3K and tyrosine kinase activities (74). IgG-mediated mast cell function is less understood than its IgE counterpart, including its direct clinical roles. Various studies show associations for FcγR-induced mast cell function in rheumatoid arthritis, Sjögren’s syndrome, multiple sclerosis, bullous pemphigoid, thyroiditis, systemic sclerosis, and glomerulonephritis (74). Thus far, much of these data are correlative. For example, mast cells and their mediators are increased in the rheumatoid synovium (75). Moreover, the results of some studies are contradictory. While there were initial reports of the inflammatory role of mast cells in experimental autoimmune encephalitis [EAE; (76)], there are contradictory reports on the role of mast cells in the disease, potentially due to differences in the severity of the EAE protocols or the mast cell-deficient mouse strains used (77, 78). While many studies have made use of W/W^v^ and W^sh^/W^sh^ c-Kit-deficient mice, newer mast cell knockout models, such as the CPA3-cre/+ mice cited above and the CPA3-Cre; Mcl-1^fl/fl^, do not have the same changes in neutrophils observed with mutations in c-Kit. Therefore, future studies may clarify the role of mast cells in IgG-associated pathologies such as multiple sclerosis and arthritis (77, 79). This area of research may provide high-impact outcomes, since IgG-induced inflammatory diseases involve pathological processes overlapping with the IgE response and may therefore be responsive to similar therapies.

### Complement Receptors

The complement system is made up of proteolytic pro-enzymes and non-enzymatic proteins that form functional complexes, co-factors, regulators, and receptors (80). Larger fragments derived from C3 and C4 regulate opsonization, phagocytosis, and immunomodulation. The smaller fragments C3a and C5a are anaphylatoxins that mediate inflammatory reactions. Anaphylatoxins can activate mast cells but, like other signals, the outcome depends on factors such as location and microenviroment (81). A few studies have examined the role of complement in mast cell activation. C3a enhances mast cell degranulation in the presence of FcγRI signaling (82), while C5a induces mast cell migration, adhesion, and mast cell mediator production (83).

### Pathogen-Associated Molecular Patterns and Toll-Like Receptors (TLRs)

Toll-like receptors are a part of the pattern-recognition receptor family interacting with a multitude of pattern-associated molecular patterns as well as host-derived damage associated molecular patterns. This family consists of 10 reported receptors (TLR 1–10) (84). Mast cells can express 9 TLRs, including TLR-1, 2, and 4–6 on the cell surface and TLR-3, and 7–9 intracellularly (85). However, some caveats are worth noting: TLR8 has not been detected on human mast cells; receptor distribution varies with mast cell location; and some receptors have only been shown at the mRNA level (86).

Toll-like receptor activation induces cytokine secretion, which can proceed through a DAP12-independent signaling cascade (87). TLR-induced cytokine profiles overlap but have some distinctions. For example, TLR-2 activation led to the production of TNF, IL-6, IL-13, IL-4, and IL-5, while TLR-4 induced TNF, IL-6, IL-13, and IL-1b (88). Interestingly, pre-exposure to TLR ligands suppressed IgE-induced mast cell responses in two mouse models, possibly by transiently reducing FcεRI expression (89, 90). By contrast, simultaneous exposure of human mast cells to various TLR ligands and FcεRI stimulation yielded increased cytokine secretion without altering degranulation (91). This is an area that warrants further study and clarification, since environmental and even laboratory exposure to allergens (e.g., house dust mite extract) is often in the context of TLR ligands.

### Compound 48/80, Substance P, and Mas-Related G Protein-Coupled Receptor-X2 (MRGPRX2)

Mas-related G protein-coupled receptor-X2 has drawn increased interest recently. Human MRGPRX2 is one of approximately 50 7-transmembrane domain proteins in the larger Mas-related gene family. It is unique in its apparently selective expression on human mast cells in the MC_TC_ subtype, outside of the dorsal root ganglion (92). While the MRGPRX gene family is restricted to humans and other primates, a mouse ortholog, MRGPRB2 has recently been described (93). Using transgene and knockout approaches, this group showed that MRGPRB2 is similarly restricted in expression to connective tissue mast cells. Both orthologs are functional receptors for compound 48/80 and the neuropeptide substance P. While of interest to those using these well-known mast cell-activating stimuli, the more important point is that MRGPRX2 (and likely its mouse ortholog) responds to other peptides and drugs. For example, MRGPRX2 binds the host defense protein LL-37 (94). Three classes of drugs have been shown to activate MRGPRX2: fluoriquinone antibiotics such as ciprofloxacin, neuromuscular-blocking drugs such as rocurinium, and the bradykinin B_2_ receptor antagonist icatibant (95). Although much remains to be done in this area, it appears that MRGPRX2 has an important role in the pseudoallergic reactions induced by some drugs. Inhibiting this receptor may therefore be clinically important.

### Chemokines and Their Receptors

Chemokines are cytokines known to induce cellular locomotion. These are particularly important in the migration of cells to areas of inflammation. All chemokine receptors described are seven-transmembrane-spanning G protein receptors (96). Mast cells have been shown to express multiple chemokine receptors, including CCR1, CCR3–5, CXCR1–4, and CX3CR1 (97). These play a significant role in directing mast cell progenitors to the tissues where they mature, a process that is altered by on-going inflammation (98–100). In addition to migration, recent studies show that chemokines can induce partial degranulation (100–102).

### Cytokines and Their Receptors

Cytokines act as messengers and modulate many functions including growth, proliferation, and migration. The importance of cytokines to mast cell biology was first shown in the 1980s, when a method to culture mast cells *in vitro* was being determined. Several groups showed that mast cells could be cultured in media from concanavalin A-activated T cells, cloned Ly + 2 inducer T cells, or WEHI-3B tumor cells. Analysis of this media showed the presence of numerous cytokines such as IL-3, IL-4, IL-9, IL-10, and nerve growth factor (1). The precise role of each cytokine is still a topic of research, partly because mast cells are a heterogenous population due to the microenvironment determining mature phenotype (103). This plasticity allows mast cells to alter their phenotype throughout their lifespan, with the phenotypic profile shaped by the cytokine and growth factor milieu they encounter (54). Our lab has been specifically interested in IL-4, IL-10, and TGF-β, which will be addressed below. In addition, the cytokines IL-33 and TSLP deserve specific attention here, due to their ability to activate mast cells and their known role in allergic disease.

IL-33 is an unusual cytokine, in that it is constitutively produced as a pro-form and localized to the nucleus of barrier cells such as keratinocytes, epithelial cells, endothelial cells, and fibroblasts. Its cleavage and release is stimulated by cell damage or inflammation, supporting its classification as an alarmin (104). Mast cells can also secrete IL-33 upon activation with signals such as IgE stimulation (105). IL-33 interacts with a receptor complex composed of T1/ST2 and IL-1RAcP (106, 107), triggering a MyD88-dependent NFκB-activating cascade resembling TLR signaling. Mast cells were among the first lineages shown to express T1/ST2 (108), 7 years before IL-33 was identified (109). IL-33 is a potent mast cell activator, eliciting survival, maturation, adhesion, and cytokine production (15, 106, 107, 110, 111). IL-33 also enhances mast cell responses to IgE (112) and IgG (113). Furthermore, IL-33 injections induce a rapid peritoneal neutrophil influx that requires mast cell-derived TNF secretion (114). IL-33 is now thought to play a significant role in mast cell-associated diseases such allergy, although precise mast cell-restricted functions are not clear and remain to be elucidated (115).

TLSP has some similarities to IL-33, including expression by epithelial and other barrier cell types and constitutive production among some lineages. In 2015, Bill Paul’s group published a ZsGreen TSLP reporter mouse, which showed TSLP expression not only in epithelial cells and keratinocytes but also in dendritic cells, basophils, and mast cells (116). TSLP secretion is induced by TLR-type signals, allergen and air irritant exposure, viral and bacterial infection, and trauma (115). TSLP interacts with a complex of TSLP-R and IL-7Rα. TSLP KO mice have reduced mast cell numbers (117), which is consistent with data showing TSLP induces mast cell proliferation through a Stat6- and MDM2-dependent pathway (117). TSLP does not induce mast cell degranulation and alone is a poor inducer of cytokine secretion. However, in combination with IL-1β + TNF, TSLP elicits the release of many cytokines, including IL-5, IL-6, and IL-13 from human CD34^+^ progenitor-derived mast cells (110, 111). It should be noted that TSLP-induced cytokine secretion has not been shown using mouse mast cells. Hence, while TSLP is clearly relevant to mast cell development and function, further studies should examine differences between mice and humans, which may be important caveats for experimental design.

## IL-4, IL-10, and TGFβ1 Regulate Mast Cell Function and Homeostasis

Regarding other cytokine effects, our lab has specific interest and experience studying IL-4, IL-10, and TGFβ effects on mast cell function and homeostasis. These cytokines augment or impair activation by the mechanisms introduced above, but do not directly induce mast cell activation alone.

### IL-4

IL-4, originally termed B cell stimulatory factor-1, is a cytokine primarily known for its role in antibody driven-allergic disease and protection from parasite infections (118). IL-4 was first discovered to induce B cell proliferation during anti-IgM stimulation and to promote isotype switching to IgG1 and IgE (119–121). In addition, Bill Paul’s lab and others showed that IL-4 elicits Th2 differentiation from naïve T cells *in vitro*, which subsequently release IL-4 in a positive feedback loop (122, 123). IL-4 signals through IL-4Rα, as part of a heterodimer containing either the common gamma chain (124) or IL13Rα (125). These receptors allow IL-4 to act on non-hematopoietic cells such as intestinal and bronchial epithelial cells and the vasculature to facilitate the protective expulsion of parasites. For details on IL-4 signaling pathways, we direct readers to reviews (126–128).

Unfortunately, IL-4 is also a major contributor to the symptoms observed with allergy and asthma (129–131). IL-4 was the first cytokine shown to be produced by mast cell lines (9), later confirmed to be secreted in response to IgE and lectin activation in human mast cells (132) as well as IL-33 in mouse mast cells (105). Mast cells also respond to IL-4, first reported to increase proliferation of mast cell lines costimulated with IL-3 by Bill Paul’s group (133). This work has been supported with data from other mast cell lines, human gut mast cells, BMMC, and in a mouse model of food allergy (9, 134, 135). Culture with IL-4 + IgE for 4–21 days has been shown to enhance FcεRI receptor expression compared with IgE alone on human cord blood, fetal liver-derived mast cells, and BMMC. It also enhances histamine, PGD_2_, and LTC_4_, and IL-5 production following IgE receptor cross-linking (134, 136–139). In addition, IL-4 differentially affected mediator release, augmenting Th2-type cytokines (IL-3, IL-5, and IL-13), and downregulating pro-inflammatory cytokines (IL-6 and TNF) in response to IgE receptor cross-linking and Gram-negative bacterial activation (137).

In contrast to its stimulatory effects on mast cells, IL-4 has been reported to suppress c-Kit expression and mast cell development in human fetal liver-derived mast cells grown in SCF in two studies (138, 140). Similarly, we showed IL-4-mediated inhibition of FcεRI and c-Kit expression on BMMC and PMC following 4–21 days of treatment, an effect dependent on Stat6 (141, 142). IL-4 suppressed IL-4, IL-5, IL-6, and IL-13 secretion induced by IgE crosslinkage, and TNF and IL-13 secretion induced downstream of SCF. Interestingly, our lab subsequently found that IL-4 increases IgG-mediated degranulation and cytokine production in mouse BMMC, involving Stat6 and increased FcγRIIIA protein expression (143). In addition to the role of IL-4 on cellular activation, we found that IL-4 induces apoptosis in developing mouse or human mast cell precursors derived from bone marrow or umbilical cord blood, respectively (144, 145).

The different pro- and anti-inflammatory effects observed by IL-4 are intriguing. Maturation, phenotype, and culture conditions likely play a role in these IL-4 responses, which we also discussed in a recent review (146). Mouse BMMC is considered less mature than human skin, human intestinal, or mouse peritoneal mast cells, which likely contributes to different experimental outcomes. For example, BMMC attain responsiveness to endothelin-1 when cultured in IL-4, while peritoneal mast cells respond to endothelin-1 without IL-4 (147). Similarly, we found that IL-4 induces apoptosis in developing mouse or human mast cell precursors (144, 145), while mature mouse and human mast cells receive survival and proliferation signals from IL-4, which also promotes the MC_T_ (tryptase-positive) phenotype in human intestinal mast cells (137). Hence, IL-4 effects on mast cells vary with stage of differentiation, with suppressive signals being most overt on developing or less mature mast cells. Future research should experimentally clarify these observations, examining cells at different maturation stages and following both IL-3 and IL-3/SCF differentiation to examine the effects of mast cell phenotype.

### IL-10

IL-10, originally termed cytokine synthesis inhibitory factor, is a homeostatic mediator in many inflammatory diseases. Secreted by macrophages, Th1 and Th2 cells, regulatory T and B cells, and cytotoxic T cells (148), IL-10 is traditionally considered an anti-inflammatory cytokine. It suppresses monocyte MHC II expression (149), dendritic cell maturation (150), and reduces inflammatory cytokine production from monocytes and neutrophils (151, 152). However, IL-10 also has stimulatory effects. It enhances B cell antibody class switching and plasma cell development (153, 154) and increases IL-2-induced proliferation and cytotoxic activity in NK cells (155). These effects correlate with clinical data, as IL-10 therapy has induced platelet loss in RA patients (156) and promoted IFNγ production in sepsis (157) and Crohn’s patients (158). In agreement with this, anti-IL-10 therapy has improved SLE measures in a clinical trial (159). While it is likely that anti-IL-10 therapy impacts many cell types *in vivo*, we have studied both the pro- and anti-inflammatory roles of IL-10 in mast cells.

Similar to IL-4, IL-10 is produced by BMMC and affects mast cell survival, proliferation, and function. IL-10 enhances IL-3-mediated growth of mouse mast cells and their progenitors (160–162). Interestingly, when co-cultured with IL-3 and IL-4, IL-10 induces BMMC apoptosis by diminishing Bcl-2 and Bcl-xL expression, in a Stat6-dependent manner (44), and induces apoptosis following IgE receptor cross-linking, a known pro-survival pathway (64).

Early work showed variable responses to IL-10 treatment. IL-10 was shown to induce BMMC expression of mouse mast cell protease (MCPT)-2 (163). Several studies showed inhibition or no change in TNF, IL-6, and histamine secretion following IgE receptor cross-linking and LPS-induced activation in HMC-1, rat peritoneal mast cells, BMMC, and human cord blood-derived mast cells (164–167). Our lab showed that 4-day IL-10 treatment inhibited FcεRI beta chain expression and IgE-induced TNF production in BMMC (168, 169). Recently, we found that while TNF is diminished, IgE-induced degranulation and secretion of other inflammatory cytokines were *increased* by IL-10 after 24-h treatment, through a Stat3–miR-155 cascade that inhibits the negative regulator, suppressor of cytokine signaling-1 (170). These effects were consistent in mouse and human mast cells and in a model of passive systemic anaphylaxis in our study, as well as a mouse model of food allergy used by Clinton Mathias’s group (160). How IL-10 suppresses TNF while enhancing other pro-inflammatory cytokines is unknown and may be important for understanding how mast cell function can be tuned. Our interpretation of these data is that while IL-10 has well-established inhibitory roles, it is not monolithic. Instead, stimulatory effects are clear from both clinical and basic research outcomes.

### TGF-β

Our group has also studied the role of TGF-β in mast cell homeostasis. Similar to IL-10, TGF-β is primarily known for its immunosuppressive effects, but pleotropic activities have been reported based on environmental and differentiation factors (171). TGF-β suppresses T cell proliferation and induces Treg differentiation (172, 173), suppresses B cell proliferation and IgG antibody class switching (172, 174), and inhibits macrophage nitric oxide release and TNF translation (175, 176).

TGF-β also alters mast cell development and function. It enhances early mast cell precursor differentiation and increases protease expression, while antagonizing survival of late stage precursors and mature mast cells (177–179). TGF-β elicits mast cell chemotaxis, but can also suppress migration toward SCF (180–182). Our lab has published that TGF-β-1, -2, and -3 inhibit the expression of FcεRI subunits, c-Kit, T1/ST2, and Fcγ receptor chains in BMMC, peritoneal mast cells, and human skin mast cells. In addition, granule formation, degranulation, and IgE-induced cytokine production were reduced by TGF-β (177, 183, 184). Recently, we found that TGF-β-1, -2, and -3 also inhibit IL-33-induced TNF, IL-6, IL-13, and MCP-1 secretion in mouse and human mast cells and suppress IL-33-induced cytokine production *in vivo* (185). Interestingly, a mouse model of lung inflammation suggests TGF-β enhances LPS-induced mast cell IL-6 production, ultimately inducing neutrophil apoptosis and controlling neutrophilic inflammation (186).

As with IL-4 and IL-10, TGF-β effects on mast cells are altered by microenvironment and genetic background. For example, IL-4 and TGFβ1 block the expression and function of the other’s receptor, with IL-4 inhibiting TGFβ1-mediated migration and *vice versa* (187). TGF-β1 has divergent effects on C57Bl6/J versus 129/SvJ mast cells. Not only do 129/SvJ BMMCs resist TGF-β1-mediated suppression of IgE-induced cytokine secretion, these BMMCs show enhanced SCF-induced migration in the presence of TGF-β1 (180). We found that matched C57BL/6J and 129/SvJ BMMC cultures have no difference in TGF-β receptor expression, but 129/SvJ BMMC express twofold to threefold greater levels of Fyn and Stat5 proteins. Since inhibiting the Fyn–Stat5 cascade appears to be important for TGF-β1-mediated suppression, this may convey resistance. In keeping with these BMMC data, we found that human skin mast cells show considerable donor-to-donor variability in TGF-β1-mediated suppression, when measuring IgE-induced cytokine secretion. These donors also showed variable Fyn and Stat5 expression (180). An additional explanation for TGF-β1 resistance is polymorphic TGF-β receptor variation. C57BL/6J and 129/SvJ strains have known polymorphic variations in TGFβR1 between, and similar variations are tied to human cancers (188).

In summary, mast cell development, survival, and function are greatly altered by IL-4, IL-10, and TGF-β. These effects are impacted by microenvironment, since cytokines can act in opposition; by stage of differentiation, since precursors and mature mast cells can respond differently; and by genetic background, with some inbred mouse strains and human donors showing complete resistance or even opposite responses. Understanding how these signals are integrated will provide a coherent approach to mast cell-associated diseases.

## The Role of Stat5 in Mast Cell Biology

The transcription factor Stat5 is expressed ubiquitously and activated by many growth factors and cytokines, including IL-2, IL-3, GM-CSF, prolactin, erythropoietin, thrombopoietin, and growth hormone (189). Stat5 is implicated in immune homeostasis and inflammation, as mice lacking the 110-kb Stat5A/B locus had perinatal lethality and severely compromised immune systems, similar to mice lacking the proteins γc, Jak3, or IL-7R (190).

Our lab was the first to show SCF-induced Stat5-DNA binding activity in mast cells (11). Utilizing the Stat5^DN^ mouse, mutated to have truncated Stat5A and B lacking the N-terminus and able to form teramers but not dimers (191), we showed that BMMC from Stat5^DN^-expressing mice exhibited increased apoptosis and delayed cell cycle progression when cultured in either IL-3 or SCF alone (192). Specifically, we observed reduced expression of the anti-apoptotic proteins Bcl-2, Bcl-(x)l, reduced expression of the cell cycle regulators cyclin A2 and cyclin B1, reduced mitochondrial membrane potential, and greater activation of caspases-9 and -3. *In vivo*, Stat5^DN^-expressing mice were born with normal mast cell distribution, but had near complete loss of tissue mast cells by 12 weeks of age, indicating that Stat5 tetramer formation is essential for regulating mast cell survival. Furthermore, neoplastic mast cells transformed by mutant c-Kit have constitutive Stat5 activation, which can be successfully targeted to inhibit proliferation and survival (193, 194). Together, these results suggest that Stat5 plays a critical role in mast cell survival.

We later reported direct and transient Stat5 activation downstream of FcεRI-mediated mast cell stimulation (195). Stat5-deficient BMMC showed impaired immediate and late phase mediator release in response to IgE-induced stimulation, indicating a critical role for Stat5 in mast cell function. A subsequent study found that Stat5 activation depends on Fyn kinase expression, and that Fyn and Stat5 are physically associated in resting mast cells (72, 73). We also noted that Stat5 co-localizes with FcεRI in antigen-activated mast cells. Interestingly, the absence of Lyn kinase, Gab2, or SHP-1 enhanced FcεRI-mediated Stat5 phosphorylation (72, 73).

In addition to FcεRI, Stat5 has been implicated in other signaling cascades controlling mast cell function. While IL-33 does not appear to activate Stat5, Bill Paul’s group showed that IL-33-induced IL-13 secretion required IL-3-mediated Stat5 activation (196). Furthermore, IL-33 elicits a complex between its receptor, ST2/IL-1RAcP, and c-Kit, supporting the finding that IL-33 signaling is enhanced by SCF in mast cells (31). Since c-Kit is a strong Stat5 activator, Stat5 may similarly contribute to IL-33 signaling *via* this pathway. In a separate line of work, Toshio Kawakami’s group reported enhanced Stat5 activity tied to increased mast cell numbers in animal models of atopic dermatitis (AD) and lesions in the skin of AD patients (197), suggesting that Stat5 contributes to this disease phenotype.

Stat5A and B are encoded by distinct genes (198–200). While murine Stat5A and Stat5B exhibit 96% sequence similarity and a very similar expression pattern, these isoforms are not completely redundant and have unique biological activity (199). For example, Stat5A is critical for murine mast cell proliferation and survival (201). Our own targeting of Stat5A or Stat5B using siRNA has found that Stat5B has a selective influence over IgE-mediated mast cell cytokine release and SCF-induced migration, with Stat5A being dispensable (72, 73, 180). The idea that Stat5 can directly promote allergic disease is supported by recent work from Joshua Milner’s group, who linked a gain-of-function Stat5b mutation to eosinophilia, urticaria, and dermatitis (202). Although no further work was done to study the specific role and functionality of mast cells in these patients, the Stat5 mutation resulted in greater Th2 cytokine production by CD4 T cells, potentially skewing the immune response toward a Th2 response. It should also be noted that increased STAT5b activity, as observed in these patients, was associated with atopic-like skin inflammation that typically involves mast cell activation. Collectively, these findings show that Stat5 is one of the central factors controlling mast cell survival and function. With evidence that Stat5 can be targeted pharmacologically, we see this as a productive avenue for addressing mast cell-associated diseases.

## Therapeutics Targeting Mast Cells

Due to the variety of inflammatory diseases in which mast cells participate, targeting mast cell function and survival can be broadly effective. This section will briefly discuss current and potential mast cell-directed therapies. We also refer the reader to several recent reviews focused on this topic (203–206).

There are several therapeutics on the market that are FDA approved for allergic disease or asthma, targeting mast cell activation and/or mediators. These include H1 inhibitors that prevent histamine signaling (207) and antagonists of leukotriene synthesis or signaling *via* the CysLT1 receptor (208). While these drugs are effective in targeting select mediators, broader inhibition of the many mast cell-derived inflammatory factors can be needed for clinical efficacy. We should note here that the newer generation antihistamines have better binding affinity for histamine receptors, reduced adverse side effects, and often have mast cell stabilizing and anti-inflammatory properties in addition to their effects on histamine, which may provide better relief for patients (209). Corticosteroids such as dexamethasone are effective inhibitors of mast cell activation and a proven treatment for mast cell-associated diseases (210, 211). In fact, we recently found that dexamethasone inhibits not only IgE- but also IL-33-mediated mast cell function (212). However, steroid medications have many adverse effects and reduced efficacy in viral-induced exacerbations, asthmatics who smoke, and in more severe forms of the disease (213, 214). The mast cell stabilizer disodium cromoglycate is approved for some mast cell proliferative and activation diseases (215), although the mechanism of action is poorly understood (216), effects are slower than drugs like antihistamines, and there is evidence that mast cell stabilizers are less efficacious than inhaled corticosteroids for asthma (209, 217). More recently, a humanized anti-IgE antibody, omalizumab, has been developed to prevent binding of circulating IgE to FcεRI and the downstream effects of cross-linking (218, 219). Omalizumab is a common and preferred treatment for chronic urticaria (220, 221) and shows efficacy for the treatment of asthma (218, 219); however, like many drugs, there appear to be responders and non-responders (222). These drugs represent both progress toward suppressing mast cell function and shortcomings supporting further development.

### Repurposing FDA-Approved Drugs

There are several FDA-approved therapeutics with potential to treat diseases caused or exacerbated by mast cell activation (Table 1). For instance, the tricyclic antidepressant doxepin was found to be a potent H1 receptor inhibitor and is prescribed to treat chronic urticaria and AD (223). The repurposing approach to targeting mast cells represents a potentially rapid avenue for clinical progress and includes several drug classes.

**Table 1:** Potential therapies for mast cell-associated diseases.

Drug	Target	Disease	Comments	Status	Reference
Imatinib	BCR-Abl/c-Kit	Asthma		FDA-approved for mastocytosis lacking D816V	(224–226)
Masitinib	c-Kit, possibly Fyn, and Lyn	Asthma		In clinical human trials	(227–230)
R112	Syk	Allergic rhinitis	Early stage results promising	In clinical trials	(231, 232)
Idelalisib	PI3K	Allergic rhinitis	Early stage results promising	In clinical trials	(233)
Statins	HMG-CoA reductase	Asthma	Mixed results, possibly due to varied responses on different genetic backgrounds	Off-label use of drug approved for hypercholesterolemia	(234–237)
Etanercept	TNF	Asthma	Early stage trials for severe asthma. Safety concerns noted	Off-label use of drug approved for use in rheumatoid arthritis	(238, 239)
siRNA, morpholino oligonucleotides, CRISPR/Cas9	Many possible: FcεRI, c-Kit, ST2, tryptase, chymase	Mast cell-associated pathology	Most work is in the conceptual stage, with some *in vivo* rodent studies done	Morpholino-based approach for Duchenne muscular dystrophy is approved	(93, 240–244)

*This table includes the name of the drug, the known targets, and diseases for which research has currently been conducted using the drug. In addition, important comments on the drug and FDA approval and/or clinical trials progress are noted*.

#### Kinase Inhibitors

Imatinib is a chemotherapeutic agent designed to target the BCR–ABL tyrosine kinase common in chronic myeloid lymphoma (224). Although imatinib was designed to target the ABL tyrosine kinase domain, it also inhibits c-Kit kinase activity. Due to this off-target effect, imatinib has been used to treat mastocytosis cases lacking the c-Kit D816V mutation (225). More recently, imatinib has been tested in a clinical trial to treat severe refractory asthma. Patients treated with imatinib had reduced airway hyperresponsiveness and decreased serum tryptase levels compared to placebo (226), supporting broader use of this drug. Unlike imatinib, masitinib was designed as a c-Kit kinase inhibitor and has been used therapeutically to treat canine mast cell tumors (227, 228). It has since entered clinical trials for human mastocytosis and asthma. In a phase 2a clinical trial, masitinib improved the quality of life in 14 of 25 mastocytosis patients for at least 60 weeks (229). It is also in a phase 3 clinical trial to treat severe and persistent asthma in conjunction with corticosteroids (227). An additional kinase inhibitor capable of suppressing c-Kit, toceranib phosphate, is being tested in canines (236). Kinase targeting in mast cell-associated diseases is not limited to c-Kit. The Syk kinase inhibitor R112 has shown mixed results for allergic rhinitis in two trials (231, 232). Similarly, the phosphatidyl inositol 3′-kinase inhibitor idelalisib has shown progress in a phase 1 trial for allergic rhinitis (233). These studies collectively support the approach of targeting kinases activated early in signaling cascades controlling mast cell function.

#### Statins

Statin drugs are HMG-CoA reductase (HMGCR) inhibitors designed to reduce cholesterol synthesis (234). These drugs are primarily approved to treat hypercholesterolemia and reduce cardiovascular disease, but have been beneficial in asthma and atopic diseases, albeit with mixed results. For example, a 1-month trial of simvastatin as a monotherapy for asthma showed little benefit (245), but subsequent studies demonstrated positive effects. Most have employed simvastatin or atorvastatin as an adjuvant therapy. Simvastatin has been shown to decrease eosinophils, improve lung function, and promote Treg development in mild asthmatics (235, 246). Similarly, atorvastatin decreased sputum inflammatory cytokine levels, suppressed LTB_4_ production, and improved quality of life scores among mild-to-moderate asthmatics (247–249). A retrospective study found that among severe asthmatics, statins in combination with inhaled therapies had better asthma control in comparison to patients who were not currently taking statins (236). Despite these encouraging findings, meta-analysis studies show that the overall effects of statins in asthma are at the best modest (249–251).

These conflicting outcomes prompted us to study statin effects on mast cells. Our lab found that one drug in particular, fluvastatin, blocked FcεRI-mediated mast cell activation in human and mouse mast cells and reduced passive systemic anaphylaxis in mice (237). However, these effects showed strong genetic influences: BMMC derived from C57BL/6J mice were most sensitive, BALB/c showed intermediate responses, and 129/SvJ mice were completely resistant. Human mast cells cultured from multiple donors showed similar variation. Our data showed that statin resistance was not tied to HMGCR coding polymorphisms, but did correlate with drug-induced HMGCR upregulation. More importantly, geranylgeranyl transferase (GGT), downstream of HMGCR in the cholesterol pathway, appears to be critical for FcεRI-mediated function. These findings suggest that statin efficacy in mast cell-associated disease might be predicted by measuring drug-induced HMGCR expression, and that targeting GGT may be a better means of disrupting mast cell function.

### Targeting Gene Expression

Another promising means of inhibiting mast cell activation is by selectively suppressing gene expression, including FcεRI, c-Kit, histadine decarboxylase, or other mast cell receptors and mediators. There are several approaches that hold promise, with two decades of clinical trials supporting progress. Morpholino oligomers bind mRNA and either block translation or modify pre-mRNA splicing and induce exon skipping (252). This approach has recently been demonstrated in mice showing that morpholinos targeting the FcεRI β-subunit decreased IgE receptor expression and function on mast cells and basophils (240) and was beneficial in treating a mouse model of allergic dermatitis. Morpholino-based therapy is approved for Duchenne muscular dystrophy (243, 244), suggesting that this approach can succeed.

siRNAs are another gene targeting tool, explored in various clinical trials and on the cusp of FDA approval (253). Similar to morpholinos, siRNAs base pair with mRNAs, inhibiting translation or decreasing half-life. Several studies have used siRNAs to diminish mast cell activation or mediator production *in vitro* (93, 241, 242, 254). A critical step for any nucleotide-based approach is customizing targeting and improving cellular uptake. Several approaches are under study, including nanoparticles and lipid-based carriers (255). Progress in this area may also come from excitement surrounding the CRISPR/Cas9 system. Although less vetted in clinical trials than morpholinos and siRNA, the specificity and efficacy of CRISPR/Cas9 elicits great hope for molecular-based therapies in many fields, including mast cell-associated disease.

### Potential Targets for New Inhibitors

Although several mast cell mediators and receptors are targeted by existing therapies, others warrant consideration. Tryptase is a mast cell protease that is expressed by all mast cells and contributes to inflammation in atopy and several autoimmune diseases by causing smooth muscle contraction and fibrosis (205, 206). Several beta-tryptase inhibitors have entered clinical trials, with APC 366 moving as far as phase 2a for asthma. However, issues with target selectivity, formulation, and reproductive toxicity have thus far prevented these inhibitors from gaining FDA approval (256). There is potential for antibodies targeting tryptase to be used for mastocytosis and atopic diseases (257). Chymase is another pro-inflammatory protease made in abundance by mast cells with the potential to be targeted by chemical inhibitors or antibodies (258). Several small molecule inhibitors such as ONO-WH-236 have been developed to selectively inhibit chymase, but none of these drugs have been clinically tested in patients with mast cell-associated diseases (205, 259).

As stated above, IL-33 activates mast cells (106, 107, 260) and is elevated in patients with asthma and AD (106, 107, 261, 262). Since it also activates Th2 cells, targeting IL-33 or its receptor ST2 could be effective. An anti-ST2 human monoclonal antibody, MSTT1041A, is in phase 2 of clinical trials to treat severe asthma (http://Clinicaltrials.gov, NCT02918019). In addition, an anti-IL-33 monoclonal antibody is in a phase 2 clinical trial for peanut allergy (http://Clinicaltrials.gov, NCT02920021) and also in phase 2 trials for AD (EU Clinical Trials Register number 2016-002539-14). With varied mechanistic targets for mast cell activation and different classes of therapeutics currently being studied, there is reason to be hopeful for progress in this area.

## Conclusion

By demonstrating that mast cells produce a Th2-type profile of cytokines, Bill Paul’s group allowed those of us fortunate to work in this field to expand our horizons and our definition of what the mast cell is. The current view is of a long-lived innate immune cell with considerable plasticity that responds to its microenvironment through a range of surface receptors, allowing the mast cell to greatly alter the course of immunity. It is therefore an ideal target for therapeutic intervention and a lineage that still yields novel insights, more than a century after its discovery.

## Author Contributions

All authors wrote and edited the manuscript.

## Conflict of Interest Statement

The authors declare that the research was conducted in the absence of any commercial or financial relationships that could be construed as a potential conflict of interest.
